# Hyperdopaminergic States Bias Explorations–Exploitation Trade‐Off in Foraging and Impair Survival

**DOI:** 10.1111/ejn.70669

**Published:** 2026-09-08

**Authors:** Wanhao Chi, Wenqin Fu, Cristianne R. M. Frazier, Liwen Xu, Jeff A. Beeler, Xiaoxi Zhuang

**Affiliations:** ^1^ Department of Neurobiology The University of Chicago Chicago Illinois USA; ^2^ Committee on Neurobiology The University of Chicago Chicago Illinois USA

**Keywords:** dopamine, feeding, fitness, learning, motivation

## Abstract

Dopamine plays central roles in reinforcement learning and motivation, allowing animals to evaluate energetic costs and gains for efficient foraging. However, how genetic variation in dopaminergic function influences survival remains poorly understood. Here, we used *Drosophila melanogaster* , an established model for studying fitness, to investigate the effects of hyperdopaminergic state on survival in energetically limited environments. We used the well‐characterized dopamine transporter mutant *fumin* (*DAT* ^*fmn*^), thermogenetic activation and pharmacological manipulations to alter dopamine signalling. In free‐feeding environment, *DAT* ^*fmn*^ mutants displayed normal food consumption and survival. However, in a foraging environment with energetic scarcity, *DAT* ^*fmn*^ mutants exhibited reduced meal frequency and impaired survival compared with controls. Dopaminergic contribution to survival was further supported by data from genetic rescue, pharmacological rescue and thermogenetic activation. Because dopamine modulates both learning and exploration‐exploitation bias, we dissociated these processes experimentally. Amphetamine treatment during the post‐learning phase, but not during learning, impaired survival, indicating that exploration–exploitation bias after learning, rather than impaired learning, accounts for the observed phenotype. Moreover, *DAT* ^*fmn*^ flies showed increased exploratory activity and reduced exploitation of known food sources. These results demonstrate that hyperdopaminergic state disrupts the balance between exploration and exploitation, thereby compromising survival under conditions of limited energy availability.

## Graphical abstract

Food scarcity causes lethal foraging due to foraging energy cost exceeding foraging energy gain. Hyperdopaminergic states (hypDA) increase lethal foraging due to increased energy cost and decreased energy gain under food scarcity conditions.

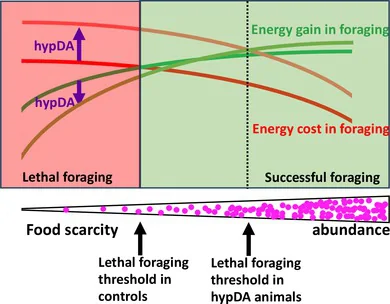

**Abbreviations**
AMPT: alpha‐methyl‐p‐tyrosine
DAT: dopamine transporter
fumin: fmn
PBS: phosphate buffered saline
PI: preference index

## 1 Introduction

Foraging requires evaluating the energetic costs and gains of each choice (Glimcher 2002; Pearson et al. 2014; Sweis et al. 2018; Schwarz et al. 2022; Doren et al. 2023). When food is scarce, survival depends on balancing the energy obtained from food acquisition with the energy expended to locate it. The relative effectiveness of different strategies, such as exploiting known food sources versus exploring new ones, varies with environmental conditions. In energy‐rich environments, exploration and energy expenditure can be advantageous, whereas in energy‐poor environments, energy conservation and exploitation of prior knowledge are essential. Although neural mechanisms underlying reward valuation and economic decision‐making have been extensively studied (Glimcher 2002; Pearson et al. 2014; Koralek and Costa 2021; Lloyd et al. 2023; Sukumar et al. 2024), their impact on adaptive fitness and survival in different energetic environments remains largely unexplored.

In mammals, the neurotransmitter dopamine is central to reward processing and value‐based decision‐making. Phasic dopamine signalling supports reinforcement learning (Dayan and Balleine 2002; Schultz 2002; Wise 2004; Gan et al. 2010; Day et al. 2011; Chowdhury et al. 2013; Eshel et al. 2015; Gershman et al. 2024; Masset et al. 2025), whereas baseline dopamine levels have been found to regulate motivation and response vigour (Cagniard et al. 2006; Berridge 2007; Niv et al. 2007; Robbins and Everitt 2007; Salamone et al. 2009). Our previous studies in mice suggest that baseline dopamine determines how reward value biases behavioural energy allocation (Beeler et al. 2010, 2012a). Elevated dopamine promotes exploration and energy expenditure, while reduced dopamine favours energy conservation and exploitation, highlighting its role in affecting post‐learning foraging strategy. Dopamine has also been implicated in coupling energy sensing to voluntary energy expenditure (de Araujo et al. 2010, 2012). However, how individual variation in dopaminergic function, such as genetic differences, affects adaptive fitness remains unknown, as survival studies in mammals are impractical.

*Drosophila melanogaster* is an ideal model for addressing this question, as survival is easily quantifiable, and gene–environment interactions can be readily tested experimentally. Dopamine's role in *Drosophila* is evolutionarily conserved, contributing to learning (Schwaerzel et al. 2003; Kaun et al. 2007; Claridge‐Chang et al. 2009; Yamagata et al. 2015, 2016; Handler et al. 2019; Jovanoski et al. 2023; Huang et al. 2024), locomotor activity (Riemensperger et al. 2011; Marquis and Wilson 2022), motivation (Burke et al. 2012), arousal (Andretic et al. 2005) and decision‐making (Zhang et al. 2007).

Here, we set up a *Drosophila* foraging paradigm modified from the capillary feeding (CAFE) assay (Ja et al. 2007; Lee et al. 2017) to examine how dopamine transporter (DAT) deficiency in the *fumin* (*DAT* ^*fmn*^) mutant, pharmacological elevation of dopamine and thermogenetic activation of dopamine neurons affect foraging behaviour and survival under varying energetic environments. We asked three questions: (1) Does elevated baseline dopamine affect survival? (2) If it does, under what environmental conditions? and (3) What changes in foraging strategy underlie altered survival? Our results show that elevated dopamine promotes exploration at the expense of exploitation of known food sources, thereby increasing energy expenditure and reducing fitness in environments of energetic scarcity.

## 2 Materials and Methods

### 2.1 *Drosophila* Strains and Maintenance

*Drosophila fumin* (*fmn*) is a mutant allele of the *DAT* gene in which insertion of a roo transposable element disrupts normal splicing at the exon 6‐intron 6 junction. This aberrant splicing introduces a premature truncation, producing a nonfunctional transporter (Kume et al. 2005). The *DAT* ^*fmn*^ mutant line was kindly provided by Dr. Jackson (Department of Neuroscience, Center for Neuroscience Research, Tufts University School of Medicine, Boston, MA, United States). The line was backcrossed to *w* ^*1118*^ (control) for five generations before use. *UAS‐DAT* (#24465), *elav‐GAL4* (#8760), *UAS‐TrpA1* (#26264), *Ddc‐GAL4* (#7009), *ple‐GAL4* (#8848) and *Trh‐GAL4* (#38388) flies were from the Bloomington Stock Center at Indiana University. Flies were maintained on standard cornmeal–yeast–molasses food from the Fly Kitchen at the University of Chicago, at room temperature (~23°C) under a 12/12‐h light/dark cycle. Since non‐virgin females may have different foraging behaviour and it is less practical to collect sufficiently large numbers of virgin females for the present study, only male flies aged 1–5 days were used for all experiments. We will interpret our data with caution because of this limitation.

### 2.2 Foraging Assays

The foraging paradigm was described in our previous study (Lee et al. 2017). Each compartment was a standard Corning 24‐well plate well (15.6 mm diameter × 18.0 mm depth) lined with 1 mL of 1% agar to maintain humidity. Calibrated glass capillaries (5 μL; VWR, Cat #53432‐706) were filled with 4% sucrose (unless a different concentration is specified in a particular experiment) containing 0.05% blue dye (Spectrum, FD&C Blue #1, Cat #FD110) for a more accurate reading. Capillary length inside the well was 0.5 cm. The number and position of capillaries were varied as described in the results. To prevent evaporation, approximately 1 μL of mineral oil was layered on top of each capillary. Flies were briefly anaesthetized with CO_2_ and transferred individually to the assay chambers. Capillaries were refilled daily after recording food consumption and survival.

### 2.3 Meal Pattern

Meal pattern analysis was performed on Day 2, when flies were acclimated to the environment and most *DAT* ^*fmn*^ flies remained alive. The assay was adapted from Al‐Anzi et al. (2010). Food consumption by each fly was recorded hourly during the 12‐h daytime period. Any consumption event exceeding 0 μL was defined as a meal. The average meal size was calculated as total consumption over 12 h divided by the number of meals, and meal frequency was calculated as the total number of meals over 12 h divided by 12.

### 2.4 Preference Test

The preference test was performed as described previously (Jiao et al. 2007) with minor modifications. Briefly, group of 20 flies were starved overnight on 1% agarose and then transferred to 96‐well plates containing alternating wells of two test mixtures. Each mixture consisted of either 4% or 0.5% sucrose in 1% agar, supplemented with blue or red food dye (Spectrum, FD&C Red #40, Cat #FD140). Flies were allowed to feed for 90 min at room temperature in the dark, after which plates were frozen at −20°C. The numbers of flies with blue (*N* ^B^), red (*N* ^R^) or purple (*N* ^P^) abdomens were determined by visual inspection, and ambiguous cases were verified by gut dissection. Flies were scored as purple when the red dye intensity was between 50% and 150% of the blue dye. The preference index (PI) was calculated as PI = (*N* ^B^ + *N* ^P^/2)/*N* ^Total^ or (*N* ^R^ + *N* ^P^/2)/*N* ^Total^.

### 2.5 Survival Under the Free‐Feeding and Starvation Conditions

A group of 20 flies was transferred to culture vials containing either 4% sucrose or 0% sucrose in 1% agar. Mortality was recorded daily.

### 2.6 Food Consumption Under Free‐Feeding Conditions

A group of 25 flies were starved overnight and then allowed to feed for 30 min on 1% agar containing 4% sucrose and 1% blue dye at room temperature in the dark. After feeding, flies were frozen on dry ice, homogenized in 500 μL phosphate buffered saline (PBS) and centrifuged at 13,000 rpm for 25 min. The supernatant was transferred to a new tube, centrifuged again under the same conditions, and the final supernatant was used for spectrophotometric analysis at 630 nm. The absorbance measured from flies fed with regular food was subtracted from that of dye‐fed flies, and the resulting value represented the amount of food consumed.

### 2.7 Locomotor Activity

Locomotor activity was monitored using the DAM2 Drosophila Activity Monitor (TriKinetics Inc., MA), as described previously (Chi et al. 2022). Individual flies were housed in glass vials (5 × 65 mm) containing 4% sucrose in 1% agar at one end, with the other end sealed with cotton. Activity was recorded continuously, and data from Day 4, after acclimation, were used for analysis.

### 2.8 Activity Distribution

Each assay well was divided into two semicircles by a line drawn on the lid with the capillary positioned along the midpoint of one semicircle's vertical radius. Fly behaviour was recorded for 30 s each trial, one trial per hour between 10:00 AM and 3:00 PM. Activities were categorized as follows: (1) ‘on target’, if the fly stayed on the capillary side of the lid, (2) ‘near target’, if the fly stayed on the non‐capillary side of the lid and (3) ‘off target’, if the fly stayed on the sidewall or floor of the well. The percentage of each activity type was calculated for each fly and normalized to the corresponding area.

### 2.9 Thermogenetic Activation of Dopamine and Serotonin Neurons

Thermogenetic activation was performed using the temperature‐sensitive cation channel TrpA1 (Hamada et al. 2008). *Ddc‐GAL4* (#7009), *ple‐GAL4* (#8488) and *Trh‐GAL4* (#38388) lines were each crossed to *UAS‐TrpA1* (#26264). Male flies were transferred to foraging assay and maintained at 31°C for thermogenetic activation. Capillaries were refilled daily after recording food consumption and survival.

### 2.10 Amphetamine Treatment

Control flies were briefly anaesthetized with CO_2_ and transferred to the 1‐capillary assay. Capillaries were filled with either 4% sucrose or sucrose containing 10‐, 11‐ or 12‐mM amphetamine (Sigma, Cat #A5880). Three conditions were tested: (1) amphetamine throughout Days 1–8, (2) 4% sucrose during Days 1–3 followed by amphetamine during Days 4–8 and (3) amphetamine during Days 1–3 followed by 4% sucrose during Days 4–8. Capillaries were refilled daily with the corresponding solution after recording food consumption and survival.

### 2.11 Alpha‐Methyl‐p‐Tyrosine (AMPT) Treatment

AMPT treatment was used to inhibit dopamine synthesis. Both *w* ^*1118*^ and *DAT* ^*fmn*^ flies were pretreated with 0.25 mM AMPT in 4% sucrose for 96 h. Flies were then transferred to the foraging assay with 0.2 mM AMPT in 4% sucrose in capillaries. Control groups received 4% sucrose without AMPT during both pretreatment and assay conditions.

### 2.12 Quantification and Statistical Analysis

Statistical analyses were performed in R (version 4.5.1). Details of the statistical tests, sample sizes, significance levels and multiple‐comparison corrections are provided in the figure legends.

Blinding was not used. Genotype difference and treatment difference were very dramatic and obvious. In addition, one main person in the lab worked on all the drug treatment experiments, and those experiments required a highly skilled person to perform them at the same time every day consistently.

## 3 Results

### 3.1 Hyperdopaminergic *DAT* ^*fmn*^ Mutant Flies Consume Less and Exhibit Lethality in a Food Scarcity Foraging Paradigm

**FIGURE 1:**
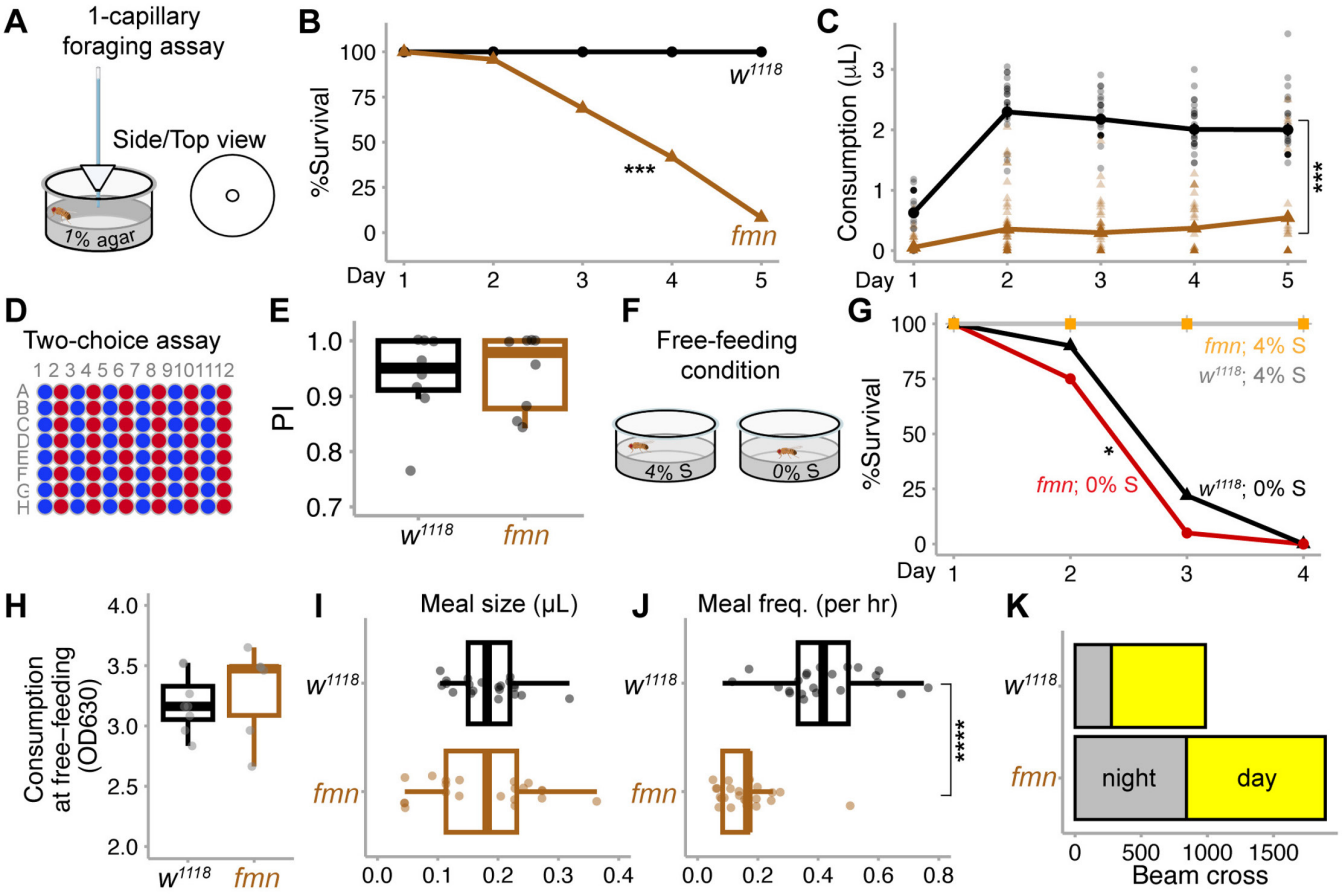
A foraging environment reveals an environment‐dependent lethal phenotype in hyperdopaminergic *DAT* ^*fmn*^ mutant flies. (A) Schematic of the foraging assay (side and top views). A single capillary containing 4% sucrose and 0.05% blue dye was inserted through the top of each cylinder chamber; 1% agar was placed at the bottom to maintain humidity. (B, C) Survival and food consumption of *w* ^*1118*^ (control) and *DAT* ^*fmn*^ (*fmn* was used in all the figures for simplicity) flies in the foraging assay. *n* = 24–48. ***p < 0.001, log‐rank test for survival; repeated‐measure ANOVA for consumption. (D) Schematic of the two‐choice assay pairing 4% sucrose with either blue or red dye. (E) Preference index (PI) of *w* ^*1118*^ flies in the two‐choice assay. *n* = 4 per genotype per dye. (F) Schematic of the free‐feeding condition with 4% or 0% sucrose in 1% agar (4% S or 0% S) at the chamber bottom. (G) Survival of *w* ^*1118*^ and *DAT* ^*fmn*^ flies under free‐feeding conditions. *n* = 100 per genotype per condition. *p < 0.05, log‐rank test, survival of *DAT* ^*fmn*^ under 0% sucrose compared with *w* ^*1118*^ flies in the same condition. (H) Food consumption of *w* ^*1118*^ and *DAT* ^*fmn*^ flies in the free‐feeding condition. *n* = 6–9 groups per genotype. (I, J) Average meal size and meal frequency of *w* ^*1118*^ and *DAT* ^*fmn*^ flies in the foraging assay. *n* = 23–24. ****p < 0.0001, unpaired *t*‐test. (K) Locomotor activity of *w* ^*1118*^ and *DAT* ^*fmn*^ flies. *n* = 8 per genotype.

We previously established a food scarcity foraging paradigm with limited food availability for adult flies (Lee et al. 2017), in which the only food source is a single capillary containing 4% sucrose (Figure 1A). To validate that this type of foraging is sensitive to manipulations of food scarcity, we tested conditions in which 1% or 0.5% sucrose was freely available at the bottom of the well in addition to 4% sucrose provided in a capillary. Under these conditions, capillary consumption was significantly reduced (Figure S1), indicating that exploitation of the capillary food is diminished when food is readily available everywhere.

To assess the effect of dopamine dysfunction on foraging behaviour and fitness, we tested *DAT* ^*fmn*^ mutants, which lack functional DATs and consequently exhibit enhanced extracellular dopamine signalling (Kume et al. 2005; Ueno and Kume 2014). In the foraging environment, most *DAT* ^*fmn*^ mutants died by Day 5, while control (*w* ^*1118*^) flies survived well (Figure 1B). Consistently, *DAT* ^*fmn*^ flies consumed significantly less than controls (Figure 1C).

To rule out the possibility that reduced consumption in *DAT* ^*fmn*^ flies was due to sucrose aversion, we performed a two‐choice preference assay (Figure 1D). Comparable preference for 4% sucrose between *DAT* ^*fmn*^ and control flies was observed (Figure 1E). We further tested control and *DAT* ^*fmn*^ flies in a free‐feeding environment, where sucrose was readily available (Figure 1F). Both genotypes survived well (Figure 1G) and had similar consumption (Figure 1H). Thus, lethality in *DAT* ^*fmn*^ mutants was specific for the food scarcity foraging environment.

### 3.2 Reduced Meal Frequency Underlies the Low Consumption of *DAT* ^*fmn*^ Flies in the Food Scarcity Foraging Paradigm

Adult flies eat intermittently, and the total consumption depends on meal size and frequency (Al‐Anzi et al. 2010). To determine whether *DAT* ^*fmn*^ flies' low consumption was due to smaller meals or reduced frequency, we measured meal size and frequency on Day 2 of foraging, when flies were acclimated to the environment and most of the *DAT* ^*fmn*^ flies were still alive. *DAT* ^*fmn*^ mutants displayed normal meal size but significantly fewer meals than controls (Figure 1I,J), suggesting that elevated baseline dopamine does not affect meal termination mechanisms but reduces meal initiation frequency. It is worth noting that the meal data were collected during the daytime. There is a possibility that the meal frequency in *DAT* ^*fmn*^ flies is higher than that in control flies at night since *DAT* ^*fmn*^ flies are also active at night (Figure 1K) (Kume et al. 2005). Nevertheless, since the meal size is unchanged in *DAT* ^*fmn*^ flies, we attributed the low consumption to their reduced meal frequency.

### 3.3 Genetic and Pharmacological Rescue

To test whether the foraging deficiency of *DAT* ^*fmn*^ flies results from loss of DAT function, we generated genetic rescue flies by re‐expressing DAT in *DAT* ^*fmn*^ flies using the UAS/GAL4 binary system (Brand and Perrimon 1993). Neuronal re‐expression driven by *elav*‐GAL4 promoter partially restored survival (Figure s2A) and rescued Day 1 consumption (Figure S2B).

In addition, we also tested whether pharmacologically reducing dopaminergic tone could improve survival of *DAT* ^*fmn*^ flies in the food scarcity foraging paradigm. Inhibition of dopamine synthesis with the tyrosine hydroxylase inhibitor AMPT partially rescued *DAT* ^*fmn*^ survival, delaying the majority of deaths from Day 4 to Day 5 (Figure S2C), consistent with elevated dopamine contributing to the phenotype. However, AMPT did not alter food consumption (Figure S2D).

Together, these genetic and pharmacological data support a role of hyperdopaminergic tone in the lethal foraging phenotype. The incomplete rescue further suggests that chronic dysregulation of dopamine signalling across development and adulthood in *DAT* ^*fmn*^ flies exerts effects that cannot be fully reversed by DAT re‐expression or transient inhibition of dopamine synthesis.

### 3.4 Hyperdopaminergic States During Post‐Learning Foraging, but not During Learning, Causes Lethal Foraging

The *DAT* ^*fmn*^ mutant flies are hyperactive and have an increased metabolic rate; consequently, they die earlier under starvation (Figure 1G,K) (Ueno et al. 2012). However, they consume similarly in the free‐feeding environment (Figure 1H) and have a normal lifespan (Kume et al. 2005). Thus, their reduced meal frequency in the foraging environment is likely due to their behavioural deficits in that environment.

In the foraging environment, control flies showed increasing consumption from Day 1 to Day 2 (Figure 1C), suggesting learning of the capillary location. To confirm it, we trained control flies for 2 days in the foraging chamber and then relocated the capillary in one group on Day 3 and left the capillary position unchanged in the control group (Figure S3A). Consumption dropped immediately in the capillary‐relocated group but recovered by Days 5–7 (Figure S3B). Such changes in consumption were due to the capillary location change because consumption in the control group remained stable across days. Together, these results confirm that efficient foraging in control flies depends on initial spatial learning followed by a stable post‐learning foraging phase.

**FIGURE 2:**
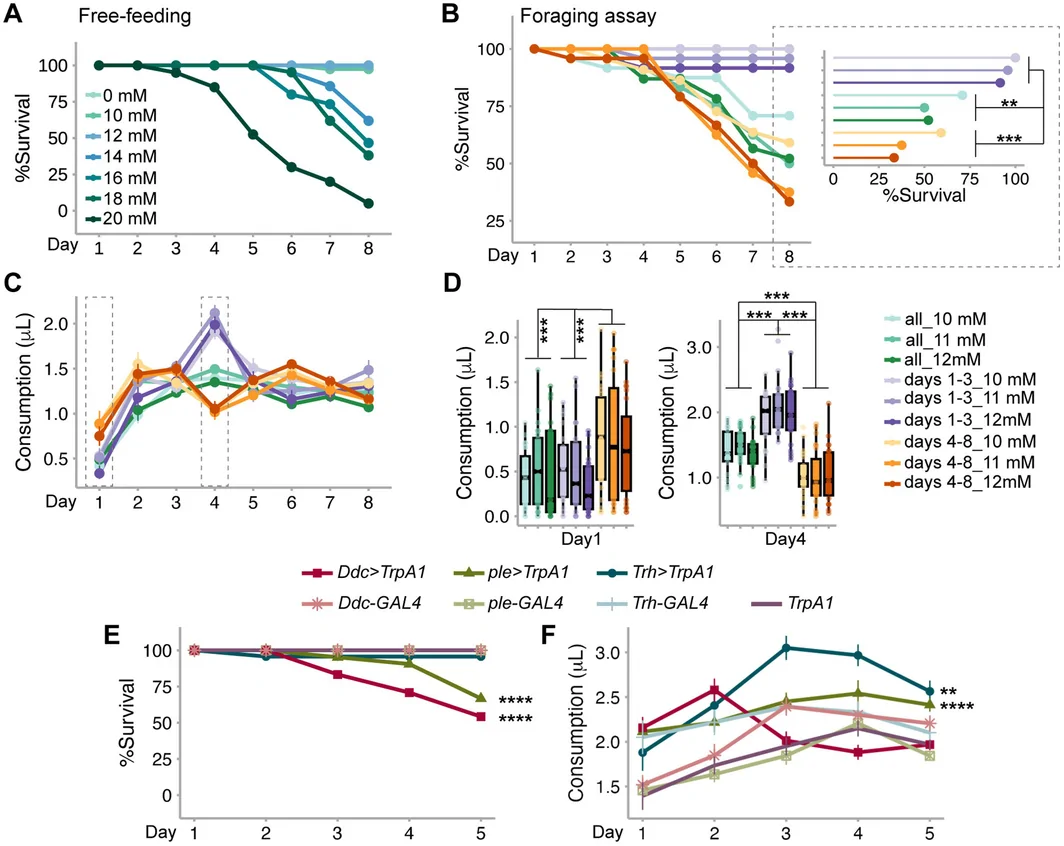
Lethal foraging caused by amphetamine and thermogenetic activation of dopamine neurons. (A) Survival of *w* ^*1118*^ flies treated with different doses of amphetamine in the free‐feeding environment. *n* = 15–40 per group. (B–D) Survival and food consumption of *w* ^*1118*^ flies in the foraging assay treated with different doses of amphetamine during the learning phase (Days 1–3), performance phase (Days 4–8) or both phases (all). *n* = 22–24 per condition. **p < 0.01, ***p < 0.001, log‐rank test for survival; one‐way ANOVA with Tukey's post hoc test for consumption. Flies treated with different doses of amphetamine within each treatment scheme were combined and compared. (E, F) Survival and consumption of thermogenetic activation of dopamine and serotonin neurons in 1‐capillary assay at 31°C. *n* = 21–24 per genotype. **p < 0.01, ****p < 0.0001, pairwise log‐rank test for survival with Benjamini–Hochberg correction within each genotype; one‐way ANOVA for consumption with Tukey’s post hoc test within each genotype (see Figure S4).

To determine whether the effects of hyperdopaminergic states are due to the learning phase or the post‐learning foraging phase, we used amphetamine to acutely elevate dopamine (Pizzo et al. 2013; Wang et al. 2015; Freyberg et al. 2016). We first performed a dose–response study in the free‐feeding environment and tested a range of doses (10, 12, 14, 16, 18 or 20 mM). We found that doses above 14 mM caused lethality in free‐feeding conditions (Figure 2A), so 10–12 mM were used subsequently. Three treatment strategies were compared: (1) amphetamine during learning (Days 1–3), during post‐learning (Days 4–8) or throughout (Days 1–8). Remarkably, we found that amphetamine treatment during the learning phase affected neither consumption nor survival, which was true for all three doses (Figure 2B,C). In contrast, amphetamine treatment during post‐learning or throughout significantly affected survival. No significant survival difference was observed between the latter two groups, further demonstrating that the treatment during the learning phase had no effects. Such data also suggest that the lethal phenotype is not due to other factors such as food preference, vision or motor impairments. Notably, the lethality associated with amphetamine treatments was not accompanied by a chronic reduction in consumption in these flies, although the acute reduction was observed (Figure 2D), suggesting a less severe impairment than that in *DAT* ^*fmn*^ flies and that hyperactivity and increased energy expenditure may have contributed significantly to lethal foraging.

Since amphetamine also affects serotonin, we next used a more selective approach by expressing the temperature sensitive TrpA1 channel in defined neuronal populations. Activation of TrpA1 in dopaminergic (using *ple‐GAL4*) or in both dopaminergic and serotonergic neurons (using *Ddc‐GAL4*)*,* but not in serotonergic neurons alone (using *Trh‐GAL4*), induced lethal foraging (Figure 2E), whereas activation in either dopaminergic or serotonergic neurons alone increased Day 5 food consumption (Figure 2F and Figure S4).

These results further support a causal role for hyperdopaminergic tone in lethal foraging. However, the consumption data also suggest that TrpA1‐induced dopaminergic activation is mechanistically different from the hyperdopaminergic tone in *DAT* ^*fmn*^ flies, as it reflects acute dopamine release rather than impaired uptake.

### 3.5 The *DAT* ^*fmn*^ Mutants Display Reduced Exploitation of Known Food Source

**FIGURE 3:**
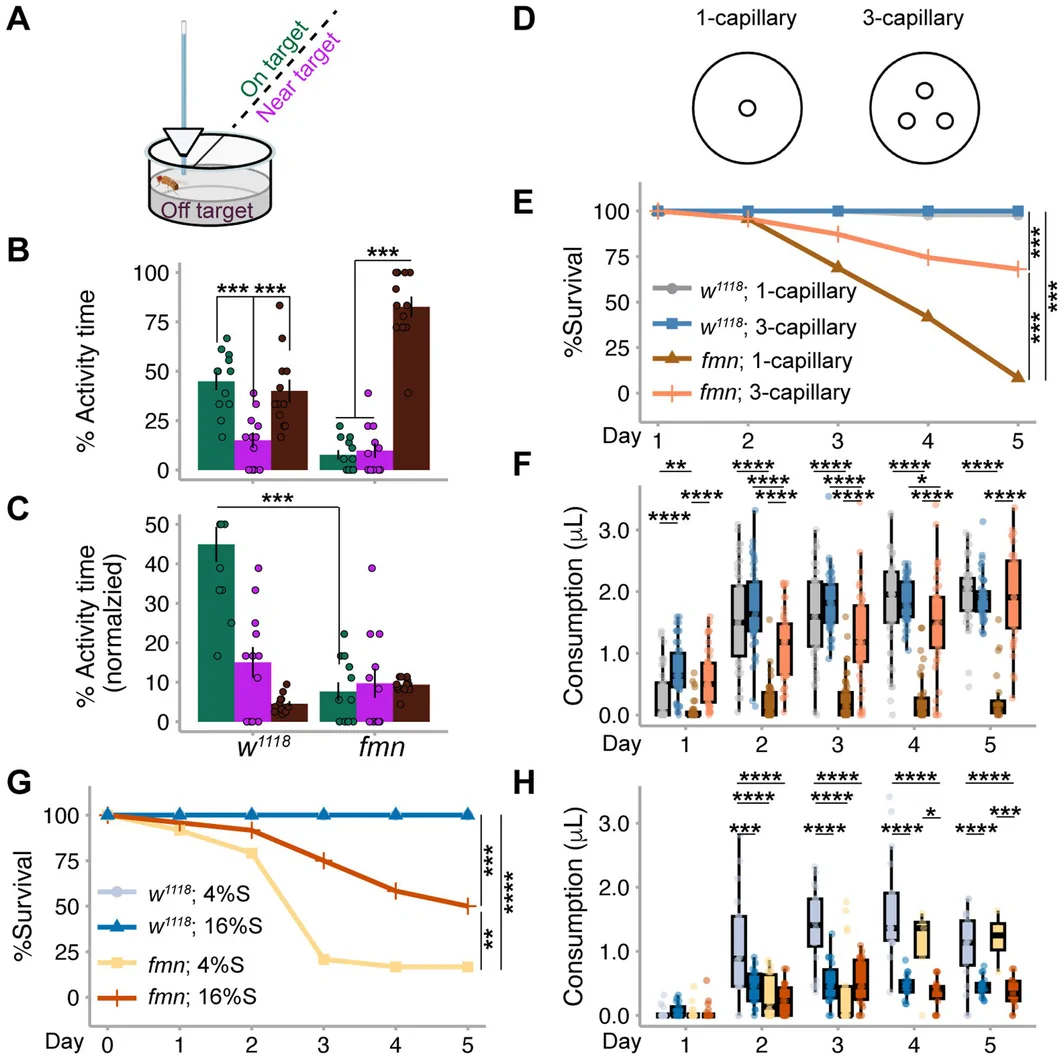
The *DAT* ^*fmn*^ mutant flies exhibited less goal‐directed behaviour, leading to environment‐dependent survival deficits. (A) Schematic illustrating how the foraging chamber was divided for assessing the spatial activity distribution. (B, C) Spatial activity distribution of *w* ^*1118*^ and *DAT* ^*fmn*^ flies before (B) and after (C) normalization to the area. *n* = 12 per genotype. ***p < 0.001, two‐way ANOVA with Tukey’s post hoc test. (D) Top view of 1‐capillary and 3‐capillary assays. (E, F) Survival and consumption of *w* ^*1118*^ and *DAT* ^*fmn*^ flies in the 1‐ and 3‐capillary foraging assays. *n* = 47–48 per group. *p < 0.05, **p < 0.01, ***p < 0.001, ****p < 0.0001, pairwise log‐rank test with Benjamini–Hochberg correction for survival; two‐way ANOVA with Tukey’s post hoc test for consumption. (G, H) Survival and consumption of *w* ^*1118*^ and *DAT* ^*fmn*^ flies in 1‐capillary assay with 4% or 16% sucrose. *n* = 20–24 per group. *p < 0.05, **p < 0.01, ***p < 0.001, ****p < 0.0001, pairwise log‐rank test with Benjamini–Hochberg correction for survival; two‐way ANOVA with Tukey’s post hoc test for consumption.

To examine choice behaviour in *DAT* ^*fmn*^ and control flies in the foraging environment directly, we divided the chamber into ‘On target’ (capillary‐containing), ‘Near target’ and ‘Off target’ regions (Figure 3A). During six 30‐s trials per fly, control flies preferentially occupied the On‐target region, whereas *DAT* ^*fmn*^ mutants spent more time Off target (Figure 3B). Since the total area of each region is different, we normalized the data accordingly. *DAT* ^*fmn*^ flies exhibited no space preference, unlike controls that maintained strong On‐target bias (Figure 3C). This indicates that elevated baseline dopamine reduces exploitation of known food source, consistent with previous reports of increased behavioural randomness in *DAT* ^*fmn*^ mutants (Sorribes et al. 2011).

The less exploitation of known food source by *DAT* ^*fmn*^ flies explains well their normal survival in the free‐feeding condition, where there is no food scarcity (Figure 1G). Indeed, when we put three capillaries instead of one in the foraging assay (Figure 3D), *DAT* ^*fmn*^ flies consumed significantly more and survived significantly better than in the 1‐capillary foraging environment (Figure 3E,F), whereas the consumption of control flies was comparable in these two environments post‐learning.

*DAT* ^*fmn*^ mutants did significantly better when we add more capillaries suggesting it is a problem of food scarcity. We also tested another condition in which there was less severe food scarcity. We increased sucrose concentration from 4% to 16% and found that *DAT* ^*fmn*^ flies survived significantly better even though they were still significantly worse than control flies (Figure 3G). While control flies adjusted their consumption quickly based on sucrose concentration, *DAT* ^*fmn*^ flies did significantly less (Figure 3H). If we compare food consumption between 4% and 16%, both *DAT* ^*fmn*^ mutants and controls consumed more volumes at 4%, suggesting that foraging is regulated by hunger in both mutants and controls even though mutants' final energy balance was not optimal. The above data also suggest that the lethal foraging phenotype is not due to impaired climbing/gravitaxis. Moreover, some *DAT* ^*fmn*^ mutants still died in the 16% sucrose condition (Figure 3G) even though *DAT* ^*fmn*^ mutants were capable of consuming more volume, which would have made them survive better (e.g., they consumed more volume in the 4% sucrose condition than in the 16% sucrose condition, Figure 3H), suggesting that lower consumption from capillary by *DAT* ^*fmn*^ mutants was not due to their inability to reach food source, rather, it is due to their biased foraging strategy and suboptimal final energy balance, and consequently less fit in environments of energetic scarcity.

To provide further evidence that reduced consumption from the capillary by *DAT* ^*fmn*^ mutants was not due to impaired climbing/geotaxis, we did one additional foraging assay in which we used the Nalgene Rapid‐Flow filter bottle top (12‐cm tall × 12‐cm diameter) instead of the 24‐well plate. It allowed us to put in 16 long capillaries instead of one short capillary in each compartment. We also added 0.5% sucrose ‘free’ food soaked in a filter paper surrounding the compartment wall in addition to the 4% sucrose in capillaries. The availability of ‘free’ food allowed *DAT* ^*fmn*^ mutants to survive well (also see Figure 1G–H) and we were able to study their consumption from capillary food for many days while we changed the capillary length to increase the climbing distance. Both *DAT* ^*fmn*^ mutants and *w* ^*1118*^ flies displayed a learning curve from Day 1 to 7, and *DAT* ^*fmn*^ mutants had overall lower consumption from capillary compared with *w* ^*1118*^ flies (Figure S5). Strikingly, when we changed the capillary length from the usual 0.5–10 cm in 2 days (from Day 7 to 9), *DAT* ^*fmn*^ mutants were not sensitive to such changes whereas *w* ^*1118*^ flies significantly dropped their consumption. These data, along with Figure 3H, again suggest that lower consumption from capillary by *DAT* ^*fmn*^ mutants was not due to their inability to climb and reach food source, rather, *DAT* ^*fmn*^ mutants had impaired ability to regulate their foraging activity based on the availability of food and associated workload (e.g., climbing distance). Moreover, their insensitivity to increased workload in foraging fits well with our published mouse data in *DAT* knockdown mice (Cagniard et al. 2006; Beeler et al. 2010, 2012a).

## 4 Discussion

The present study provides direct empirical evidence that dopamine plays a critical role in foraging strategies that affect fitness. Under conditions of energetic scarcity, survival requires both conserving energy and directing behaviour efficiently toward food acquisition. *DAT* ^*fmn*^ mutant flies failed on both counts, exhibiting excessive exploration, reduced food‐directed behaviour and reduced consumption. In contrast, *DAT* ^*fmn*^ flies had normal consumption and survived well in environment of abundance, suggesting a strong gene–environment interaction. Conversely, elevated dopamine may confer advantages in environments that demand exploration of the unknown (Beeler et al. 2010, 2012b). These data underscore the adaptive trade‐offs inherent in dopaminergic regulation and that genetic variation in dopaminergic tone likely reflects evolutionary pressure in balancing exploration and exploitation, with genetic diversity in a species ensuring population resilience to fluctuating environments.

Dopamine's role in reinforcement learning is well established across different species, including humans, non‐human primates, rodents and insects (Schultz 2002; Wise 2004; Kaun et al. 2007; Kheirbek et al. 2008; Chowdhury et al. 2013; Eshel et al. 2015; Yamagata et al. 2015, 2016; Handler et al. 2019; Gershman et al. 2024; Masset et al. 2025). However, such learning processes depend primarily on phasic dopamine fluctuations that encode prediction errors. In contrast, the *DAT* ^*fmn*^ mutation produces chronically elevated baseline dopamine levels, which, according to our data, did not impair learning in our foraging assay. Similarly, mice lacking DATs exhibited elevated baseline dopamine, hyperactivity, increased exploration and insensitivity to energy expenditure but retain normal learning ability (Cagniard et al. 2006; Beeler et al. 2010, 2012a). The consistency between fly and mouse studies suggests the role of dopamine in learning and regulating behavioural energy expenditure is evolutionarily conserved.

**FIGURE 4:**
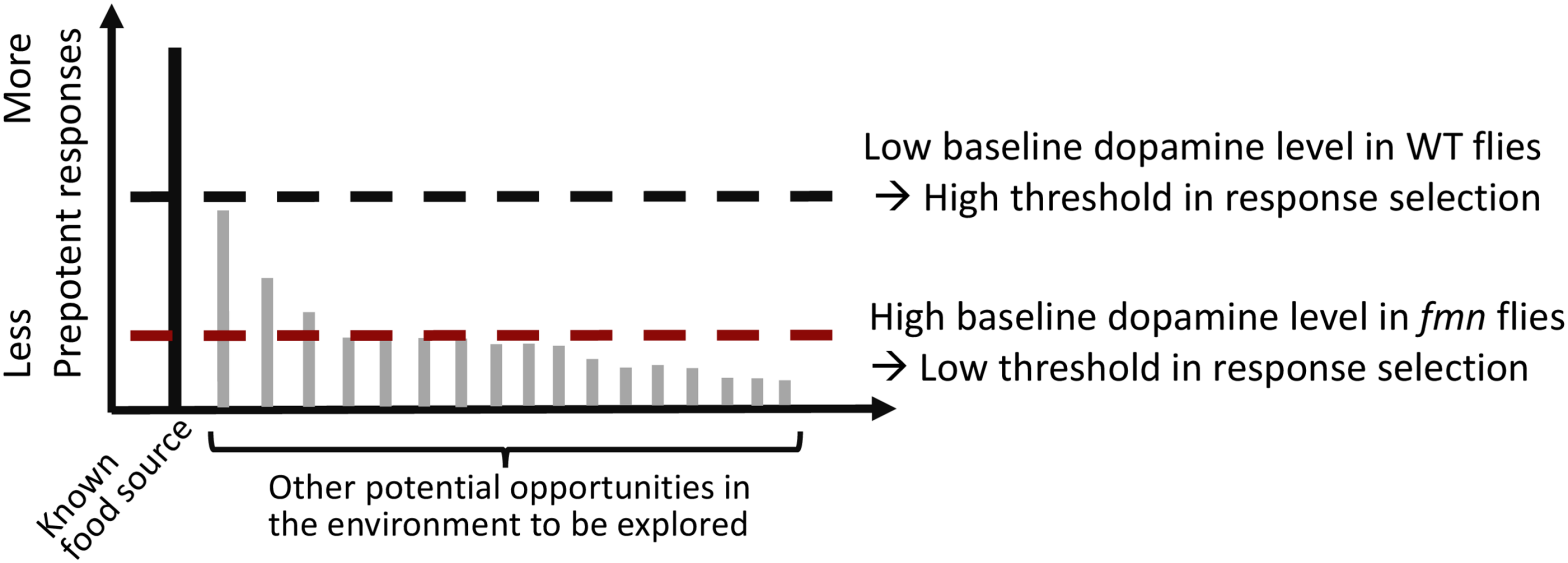
Baseline dopamine levels influence response selection and energy balance during foraging. Elevated baseline dopamine is hypothesized to reduce the threshold for response selection. In *DAT* ^*fmn*^ flies, both less prepotent responses (e.g., exploring opportunities other than the known food source) and more prepotent responses (e.g., exploiting the known food source) are selected. In contrast, control flies primarily select the most prepotent responses. As a result, *DAT* ^*fmn*^ mutants exhibit increased behavioural energy expenditure but reduced food acquisition under conditions of food scarcity.

As summarized in Figure 4, we propose that elevated baseline dopamine level lowers the threshold for response selection, increasing the probability of less prepotent responses such as exploration. In contrast, lower dopaminergic tone constrains behaviour to the most prepotent responses, such as exploiting the known food sources. The resulting excessive exploration in *DAT* ^*fmn*^ mutants leads to excessive energy expenditure and insufficient resource procurement, ultimately leading to energy deficit and death in environment of scarcity. Figure 4 model suggests a potential simple mechanism at the dopamine circuit level (e.g., dopamine could potentially increase excitability of postsynaptic neurons involved in exploration and/or decrease excitability of relevant inhibitory neurons) (Pimentel et al. 2016; Frighetto et al. 2022). This is different from a high‐level top‐down decision‐making mechanism.

Hyperdopaminergic activity has been implicated in rodent anorexia models (Klenotich et al. 2012; Kontis and Theochari 2012; Södersten et al. 2016; Beeler and Burghardt 2021; Beeler et al. 2021; Welch et al. 2021) and dopamine antagonists have been proposed to be therapeutic (Klenotich et al. 2012). Anorexia nervosa itself has been suggested to reflect maladaptation of an otherwise adaptive strategy (Guisinger 2003). Conversely, reduced dopamine function in obesity have been reported in both humans and rodents (Vucetic and Reyes 2010; Kenny 2011; Beeler et al. 2012a, 2016), which is consistent with increased energetic thrift, i.e., greater consumption and lower energy expenditure. Together, all these studies suggest that dopamine‐related genes and dopamine system functions, through regulating behavioural energy expenditure and thrift, may represent evolutionarily conserved mechanisms that play central roles in adapting energy balance to environmental conditions.

## 5 Conclusions

Our data demonstrate that hyperdopaminergic states bias the adaptive balance between exploration and exploitation, promote excessive exploration at the expense of exploiting known food sources and lead to inefficient energy procurement and reduced fitness under energetic scarcity. Hyperdopaminergic *DAT* ^*fmn*^ mutant flies had normal food consumption and survival under free‐feeding conditions but failed to survive in an energetically scarce foraging environment due to excessive exploration and reduced meal frequency. Amphetamine treatment during the post‐learning foraging phase, but not during learning, impaired survival, suggesting biased post‐learning foraging strategy rather than learning deficits as the critical factor.

## Author Contributions

**Wanhao Chi:** conceptualization, formal analysis, investigation, methodology, writing – original draft, writing – review and editing. **Wenqin Fu:** formal analysis, investigation, methodology, writing – original draft, writing – review and editing. **Cristianne R. M. Frazier:** conceptualization, writing – review and editing. **Liwen Xu:** investigation, writing – review and editing. **Jeff A. Beeler:** writing – review and editing, conceptualization. **Xiaoxi Zhuang:** conceptualization, resources, writing – original draft, writing – review and editing.

## Funding

This work was supported by National Institutes of Health, 10.13039/100000002, R01GM100768.

## Conflicts of Interest

The authors declare no conflicts of interest.

## Supporting information


**Figure S1:** Foraging is sensitive to food scarcity. (A) Schematic of free food conditions in which 1%, 0.5% or 0% sucrose in 1% agar was provided at the bottom of the well, along with in a single capillary containing 4% sucrose. (B) Consumption of *w* ^*1118*^ flies from the 4% sucrose capillary in the indicated free food conditions. *n* = 24 per group. **p < 0.01, repeated‐measures ANOVA.

**Figure S2:** Genetic and pharmacological rescue experiments support a role for hyperdopaminergic tone in lethal foraging under food scarcity. (A, B) Survival and consumption of genetic rescue and control flies in 1‐capillary assay. *n* = 48 per genotype. *p < 0.05, **p < 0.01, ***p < 0.001, ****p < 0.0001, pairwise log‐rank test with Benjamini–Hochberg correction for survival; one‐way ANOVA with Tukey's post hoc test for consumption. (C, D) Survival and consumption of *w* ^*1118*^ and *DAT* ^*fmn*^ flies in the foraging assay following treatment with vehicle (veh) or AMPT. *n* = 23–31 per group. *p < 0.05, **p < 0.01, ***p < 0.001, ****p < 0.0001, pairwise log‐rank test with Benjamini–Hochberg correction for survival; one‐way ANOVA with Tukey's post hoc for consumption.

**Figure S3:** Spatial learning is an important component in foraging. (A) Top view of the foraging assay showing the configuration with or without capillary repositioning on Day 3. (B) Food consumption of *w* ^*1118*^ flies in the foraging assay with or without capillary repositioning. *n* = 30–32 per group. *p < 0.05, unpaired *t*‐test with Bonferroni correction.

**Figure S4:** Box plots of consumption in thermogenetically activated and control flies in the 1‐capillary assay at 31°C. *n* = 21–24 per genotype. *p < 0.05, **p < 0.01, ***p < 0.001, ****p < 0.0001, one‐way ANOVA with Tukey's post hoc within each genotype.

**Figure S5:** Additional foraging assay with altered capillary length (0.5 cm vs. 10 cm). In addition to 4% sucrose in capillaries, 0.5% sucrose was freely available on the compartment walls. Increasing the capillary length from 0.5 to 10 cm did not alter food consumption by *DAT* ^*fmn*^ mutants but significantly reduced consumption by *w* ^*1118*^ flies. Each compartment contained 25 flies. *n* = 4–5 per genotype per condition. *p* < 0.01, unpaired *t*‐test for Day 9.

## Data Availability

The original dataset for main figures is available at https://doi.org/10.6084/m9.figshare.32049972.
